# Quercetin Mitigates Methamphetamine-Induced Anxiety-Like Behavior Through Ameliorating Mitochondrial Dysfunction and Neuroinflammation

**DOI:** 10.3389/fnmol.2022.829886

**Published:** 2022-02-28

**Authors:** Fengrong Chen, Jiaxue Sun, Cheng Chen, Yongjin Zhang, Lei Zou, Zunyue Zhang, Minghui Chen, Hongjin Wu, Weiwei Tian, Yu Liu, Yu Xu, Huayou Luo, Mei Zhu, Juehua Yu, Qian Wang, Kunhua Wang

**Affiliations:** ^1^School of Medicine, Kunming University of Science and Technology, Kunming, China; ^2^NHC Key Laboratory of Drug Addiction Medicine, Kunming Medical University, Kunming, China; ^3^Yunnan Institute of Digestive Disease, The First Affiliated Hospital of Kunming Medical University, Kunming, China; ^4^Center for Experimental Studies and Research, The First Affiliated Hospital of Kunming Medical University, Kunming, China; ^5^Department of Organ Transplant, The First Affiliated Hospital of Kunming Medical University, Kunming, China; ^6^Yunnan University, Kunming, China; ^7^The School of Foreign Languages, University of Shanghai for Science and Technology, Shanghai, China; ^8^Tianhua College, Shanghai Normal University, Shanghai, China

**Keywords:** methamphetamine, anxiety, mitochondrial dysfunction, oxidative stress, quercetin, neuroinflammation

## Abstract

Methamphetamine (MA) abuse results in neurotoxic outcomes, including increased anxiety and depression. Studies have reported an association between MA exposure and anxiety, nonetheless, the underlying mechanism remains elusive. In the present study, we developed a mouse model of anxiety-like behavior induced by MA administration. RNA-seq was then performed to profile the gene expression patterns of hippocampus (HIPP), and the differentially expressed genes (DEGs) were significantly enriched in signaling pathways related to psychiatric disorders and mitochondrial function. Based on these, mitochondria was hypothesized to be involved in MA-induced anxiety. Quercetin, as a mitochondrial protector, was used to investigate whether to be a potential treatment for MA-induced anxiety; accordingly, it alleviated anxiety-like behavior and improved mitochondrial impairment *in vivo*. Further experiments *in vitro* suggested that quercetin alleviated the dysfunction and morphological abnormalities of mitochondria induced by MA, *via* decreasing the levels of reactive oxygen species (ROS), mitochondrial membrane potential (MMP), and increasing the oxygen consumption rate (OCR) and ATP production. Moreover, the study examined the effect of quercetin on astrocytes activation and neuroinflammation, and the results indicated that it significantly attenuated the activation of astrocytes and reduced the levels of IL-1β, TNFα but not IL-6. In light of these findings, quantitative evidence is presented in the study supporting the view that MA can evoke anxiety-like behavior *via* the induction of mitochondrial dysfunction. Quercetin exerted antipsychotic activity through modulation of mitochondrial function and neuroinflammation, suggesting its potential for further therapeutic development in MA-induced anxiety.

## Introduction

As a highly addictive psychostimulant drug, methamphetamine (MA) abuse is an increasingly common worldwide phenomenon, resulting in significant physical, behavioral, cognitive, and psychiatric outcomes (Meredith et al., 2005; Homer et al., 2008; Glasner-Edwards and Mooney, 2014). Epidemiological studies have shown that amphetamine-type stimulants represent the most widely used illicit drugs in the world after cannabis, with ≤ 51 million global users between the ages of 15 and 64 years (Wearne and Cornish, 2018). Among abusers, 72–100% experience MA-induced psychotic reactions (Srisurapanont et al., 2003; Smith et al., 2009), and 30.2% of chronic MA users are diagnosed with anxiety (Hellem, 2016). In a recent cohort research in Australia, even more than half (60%) of the participants were classified as experiencing moderate to severe anxiety and/or depression (Duncan et al., 2021).

Human neuroimaging studies have suggested that MA users experience significant changes in multiple brain regions, including the orbitofrontal cortex, striatum, amygdala, hippocampus, and insula, which are involved in a variety of functional networks, including the salience network, limbic system, and frontostriatal circuit (Uhlmann et al., 2016; May et al., 2020; Nie et al., 2021). Further evidence has demonstrated that MA administration can evoke changes in behavior, synaptic transmission, and volume in the hippocampus (Uhlmann et al., 2016; Golsorkhdan et al., 2020), resulting in the dysregulation of neurotransmitters and their receptors in these regions (Uhlmann et al., 2016), often accompanied by oxidative stress, apoptosis, and autophagy (Huang et al., 2017). Hippocampal damage, particularly dentate gyrus (DG) and ventral hippocampus (vH), is associated with the pathogenesis of anxiety and depression (Satpute et al., 2012; Allsop et al., 2014; Baksh et al., 2021). The relation between anxiety and hippocampal activity has been subject to research for many years. Therefore, it is important to take the role of hippocampus into consideration to identify anxiety-related genes with MA treatment.

Previous studies have demonstrated that both oxidative stress and mitochondrial dysfunction may play significant affective roles in the pathology of anxiety (Chang et al., 2007; Kohno et al., 2018). Excessive dopamine induced by MA is thought to trigger the overproduction of reactive oxygen species (ROS) by the mitochondria and relevant enzymes, exacerbating neurodegenerative diseases (Shin et al., 2017), which suggested that mitochondrial functional processes may play major roles in the brain abnormalities in relation to MA-induced anxiety. Increasing experimental evidence has supported the existence of a link between mitochondrial dysfunction, brain dysfunction, and neuropsychiatric disorders (Wallace, 2017; Pei and Wallace, 2018; Filiou and Sandi, 2019). Suboptimal mitochondrial function would be vulnerable to the stress-associated depletion of the brain’s energy resources, resulting in the development of psychiatric disorders (e.g., anxiety and depression) (Morava and Kozicz, 2013). However, the mechanisms underlying the etiology of MA-induced anxiety remain poorly understood.

Research has suggested that the treatment of co-occurring psychiatric disorders, including depression and anxiety, may also be important for preventing relapses (Glasner-Edwards et al., 2010; Su et al., 2017). A substantial amount of literature has demonstrated the efficacy of both first- and second-generation antipsychotic drugs for the treatment of psychotic symptoms associated with MA-induced depression and anxiety (Shoptaw et al., 2009; Wang et al., 2016; Chiang et al., 2019). However, adverse events are frequently reported in these studies. Quercetin, which is a flavonoid-type secondary metabolite found in foods and medicinal plants, is presumed to have antioxidant, anti-inflammatory, immunoprotective, and anti-carcinogenic effects and has been found to mitigate anxiety-like behaviors in mice by modulating oxidative stress and monoamine oxidase activity (Dhiman et al., 2019), preventing antioxidant enzyme impairment, regulating serotonergic and cholinergic neurotransmission, and decreasing neuroinflammation and neuronal apoptosis (Samad et al., 2018; Kosari-Nasab et al., 2019). Quercetin has been confirmed to be safe when used as a single compound in dietary supplements in both animal and human studies, and adverse effects following supplemental quercetin intake have rarely been reported (Andres et al., 2018). Despite evidence that quercetin serves as an oxidative stress and inflammatory modulator, no research has examined the effects of quercetin on anxiety-like behaviors induced by chronic MA.

In this study, we aimed to understand the gene profiling of hippocampus of MA-induced anxious mice and determine whether quercetin intervention could mitigate anxiety-like behaviors by exploring the underlying mechanisms. We first performed RNA-seq in HIPP to identify susceptibility genes in relation in anxiety induced by MA. Then the potential underlying pathways were analyzed by Kyoto Encyclopedia of Genes and Genomes (KEGG) analyses. Subsequently, in view of the functional enrichment, we assessed the anti-anxiety effects of quercetin as a mitochondrial protector *in vitro*. Finally, we performed experiments *in vivo* and *in vitro* to further explore the role of quercetin in mitochondria and neuro-inflammation. These findings will contribute to a better understanding of the role of mitochondria in anxiety and allow for the therapeutic potential of quercetin against MA-induced anxiety to be assessed.

## Materials and Methods

### Animals and Treatment

Mice were housed with a 12-h light/dark cycle (lights on at 7:00 A.M). Behavioral testing is performed between 9:00 AM and 6:00 PM. The experimental mice were transferred to the behavioral testing room 30 min before the first trial to allow them to habituate to the room conditions. All procedures were approved by the Committee on Ethics in the Use of Animals from Kunming Medical University (CEUA no. kmmu2021227). MA was dissolved in sterile saline to a concentration of 1 mg/ml as a stock solution. Quercetin was purchased from Sigma-Aldrich Company (Sigma-Aldrich, MO, United States) and was first dissolved in polyethylene glycol (PEG, Sigma-Aldrich), at a final concentration of 50 mg/kg in 20% PEG with 0.9% saline. Adult male C57BL/6 mice which weighed from 22 to 25 g were randomly divided into three groups (*n* = 12 each group): control, MA-treated, and MA + quercetin (Q)-treated. The control group received normal saline injection intraperitoneally; the MA group received escalating MA doses, as described in a previous study (Manning et al., 2016), at 5, 10, and 15 mg/kg during the first, second, and third weeks, respectively; and the MA + Q group received escalating MA doses and quercetin treatment, administered with one dose at 50 mg/kg daily for 4 days during the first week and 5 days in the second week, as described in a previous study (Zhang et al., 2019). On the 22nd day, the open field (OFT) and Elevated Plus Maze (EPM) tests were used to examine the motor activity and anxiety levels, respectively. After behavioral testing, the animals were sacrificed immediately, and subsequent experiments were conducted.

### Conditioned Place Preference Test

The conditioned place preference test (CPP) apparatus consisted of two compartments: one had black and white striped walls, a white floor, and a black ceiling; the other had black and white checkered walls, a black floor, and a white ceiling. The two compartments were separated by a removable board. Behavioral subjects were habituated for 5 min and placed in the experimental environment for adaptation for 3 days before the CPP test.

Pre-test, conditioning, and a test were included in the MA CPP session. During the pre-test phase, the mice were placed in the middle of the conditioning apparatus and allowed to freely explore the full extent of the CPP apparatus for 15 min. The time spent in each chamber was measured. Mice that spent > 65% (> 585 s) or < 35% (< 315 s) of the total time (900 s) on one side were eliminated from subsequent CPP experiments (Zhou et al., 2019). Conditioning was conducted on mice confined to one chamber for 30 min, which was paired with an intraperitoneal (i.p.) MA injection on days 1, 3, 5, and 7, and on days 2, 4, and 6, the mice were confined to the other chamber for 30 min, which was paired with an i.p. saline injection. For the CPP test, mice were released from the middle part of the CPP apparatus and allowed to freely explore both chambers for 15 min. The CPP score was calculated by subtracting the time spent within the saline-paired side from that spent on the MA-paired side. Mouse behavior was analyzed using the ANY-maze video tracking system (Stoelting Co.).

### Open Field Test

Each experimental animal was placed in the corner of the open field apparatus (50 × 50 × 40 cm^3^, SANS Co., Jiangsu, China), which consisted of a white plastic floor and wall. The OFT performance was recorded using a video camera attached to a computer and controlled by a remote device. The total distance traveled (cm) and time spent in the center area (20 × 20 cm^2^) were recorded during a 5−min test period. After each trial, the whole open field apparatus was cleaned with 75% ethyl alcohol to efficiently remove odor to prevent any bias based on olfactory cues. The mouse behavior was analyzed using the ANY-maze video tracking system (Stoelting Co.).

### Elevated Plus Maze Test

The EPM consisted of two open arms (30 cm × 5 cm) and two enclosed arms of the same size, with 15 cm white plastic walls. The four arms were connected by a central square (5 cm × 5 cm) (SANS Co., Jiangsu, China). The arms were elevated 55 cm above the floor. Each experimental mouse was placed in the central square of the maze, facing one of the enclosed arms. The number of entries into each arm and the time spent in the open arms were recorded during a 5-min test period. When a mouse falls from the maze, the data were excluded. After each trial, all arms and the center area were cleaned with 75% ethyl alcohol, as previously described. All experimental data was collected and analyzed described.

### RNA Preparation, Library Construction, and Sequencing

Total RNA was isolated using RNA-Bee reagent, following the manufacturer’s protocol. RNA purity was determined using the NanoPhotometer spectrophotometer (IMPLEN, CA, United States), and the concentration was determined using the Qubit RNA Assay Kit (Life Technologies, CA, United States). Samples with RNA integrity values > 7.0 were used for the following experiments, which were assessed by the RNA Nano 6000 Assay Kit of the Bioanalyzer 2100 system (Agilent Technologies, CA, United States).

Sequencing libraries were prepared using the NEB Next Ultra RNA Library Prep Kit (Illumina, United States), according to the manufacturer’s recommendations, which were described in our previous research (Sun et al., 2020). In brief, mRNA was purified using poly-T oligo-attached magnetic beads, fragmentation was performed using divalent cations, and library quality was assessed on the Agilent Bioanalyzer 2100 and qPCR. The clustering of the index-coded samples was performed on an acBot Cluster Generation System using TruSeq PE Cluster Kitv3-cBot-HS (Illumina, San Diego, CA, United States). The library preparations were then sequenced on an Illumina Hiseq platform, and paired-end reads were generated.

### Quantification and Differential Expression Analysis of mRNA

As mentioned in Sun et al. (2020), the reference genome index was built, and paired-end clean reads were aligned to the reference genome using Hisat2 v2.0.5. Feature Counts v1.5.0-p3 was used to count the reads numbers. The expected number of fragments per kilobase of transcript sequence per millions (FPKM) of base pairs sequenced was calculated based on the gene length, and read counts were mapped to the gene.

Differential expression analysis (*n* = 3 per group) was performed using the DESeq2 R package (1.16.1). *P*-values were adjusted using the Benjamini and Hochberg approach for controlling the false discovery rate (FDR), and *p* < 0.05 and log_2_ fold-change > 1 were considered to be significant DEGs. DEGs enrichment in the KEGG pathways was assessed using the web tool Metascape^1^.

### Immunofluorescence Staining and Imaging

Mice were deeply anesthetized and perfused with 25 ml ice-cold PBS, followed by 25 ml 4% ice-cold paraformaldehyde (PFA) in PBS. Brains were removed and dehydrated with 15% and 30% sucrose at 4°C. The fixed brains were sliced into 30-μm-thick sagittal slices using a Leica CM1950. Slices were permeabilized in 1.2% Triton X-100 in PBS for 15 min and subject to incubation in blocking solution. Slices were incubated with primary antibodies for NeuN (1:200, Abcam), glial fibrillary acidic protein (GFAP, 1:500, Abcam) for 24 h at 4°C, followed by incubation with species-matched and Alexa Fluor conjugated secondary antibodies raised in rabbit (1:5,000, Invitrogen) for 2 h at room temperature. 4′,6-Diamadino-2-phenylindole (DAPI, 1:1,000, Invitrogen) was incubated after secondary antibody incubation for 15 min at room temperature. Slices were mounted and coverslipped using VECTASHIELD H-1000 mounting medium and scanned on a Nikon C2 confocal microscope using NIS-Element software.

### H&E Staining and Electron Microscope Imaging

The brain tissues were fixed in 4% paraformaldehyde (PFA) immediately after sacrifice. H&E staining was performed on 5 μm paraffin sections using standard H&E staining protocol which were described previously (Sun et al., 2020). The thin sections were made with an ultramicrotome, stained by OsO_4_. Electron microscopy and ultrastructural studies were performed on a transmission electron microscope (JEM-1400Flash).

### Cell Culture

Hippocampal astrocytes were prepared from C57BL/6 mouse pups under sterile conditions. Neonatal mouse pups were decapitated, after brain removal, hippocampus were separated and cut in small pieces, and incubated in trypsin solution (GIBICO) at 37°C for 20 min, DNase I (50 μl/ml) was added. Tissue pieces were washed with DMEM + 10%FBS. Cells were centrifuged at 1,000 rpm for 5 min and suspended with astrocytes medium (AM, Cell Sciences). Astrocytes were plated into poly-D-lysine (Byotime) coated 25 cm2 flasks at 1 × 10^6^ cells/flask, and grown in astrocytes medium, maintained in a humidified 37°C incubator with 5% CO2 with media exchange next day. Cells were digested with trypsin, the 3rd passages were used in all experiments.

### Measurement of ATP, Mitochondrial Membrane Potential, and Reactive Oxygen Species

Intracellular ATP was determined using a firefly luciferase-based ATP assay kit (Beyotime, Beijing, China) based on a fluorescence technique. In brief, astrocytes (1 × 10^4^) were plated in 96-wells, and appropriate drug treatments were applied for 48 h. Opaque-walled 96-well plates with culture media (50 μL) were prepared. Luminescence test solution (50 μL) was added and incubated for 30 min and then measured using a luminescence microplate reader. Mitochondria-derived ATP was measured after treatment with 300 mM iodoacetic acid (IAA, Sigma-Aldrich, MO, United States). IAA was added to half of the wells and the cells were then incubated at 37°C and 5% CO_2_ for 60 min. After 30 min, 1 mM oligomycin was added to half of the wells containing IAA and incubated for 30 min to abolish all ATP production and confirm that the ATP levels in the presence of IAA were produced by the mitochondrial ATP synthase. Subsequently, the media was removed from the wells and cellular ATP was measured using ATP assay kit (mentioned before).

JC-1 assay was conducted to analyze the MMP using the JC-1 mitochondrial membrane potential assay kit (Beyotime, Beijing, China). In brief, astrocytes (2 × 10^5^) were plated in 6-well plates and, after appropriate drug treatments, stained with 10 μM JC-1 for 20 min. JC-1 exhibits double fluorescence staining, either as red fluorescent J-aggregates (530 nm excitation/590 nm emission, as P3) at high potentials or as green fluorescent J-monomers (490 nm excitation/530 nm emission, as P2) at low potentials; Flow cytometric analysis was performed by fluorescence-assisted cell sorting (FACS) after JC-1 staining detected changes in MMP and the value was calculated. The relative proportion of red and green fluorescence was used as an index of change in membrane potential.

For ROS generation measurements, primary astrocytes were plated in 96-wells, and appropriate drug treatments were applied for 48 h. Then, cells were loaded with the ROS probe 70-dichlorodihydrofluorescein diacetate (DCFDA, 2 μM) for 40 min at RT in the dark. The mROS assay was conducted using the MitoSOX red mitochondrial superoxide indicator (Mercury Drive, Sunnyvale, CA, United States). Cells were measured by FACS at 488 nM excitation.

### Oxygen Consumption Rate Analysis

Astrocytes were plated at 7.5 × 10^4^/well in seahorse assay plates and treated with their respective treatments at 37°C and 5% CO_2_ and for 24 h. Mitochondrial oxygen consumption rate (OCR) was measured by extracellular flux (XF) assay (Seahorse XFp analyzer, Agilent Technologies, Santa Clara, CA, United States), according to manufacturer’s procedures. Briefly, cells were incubated in a CO_2_-free environment for 1 h, and OCR was measured every 3 min for the next 90 min. First, OCR was acquired in basal conditions (20 mM glucose), followed by in the presence of 1.5 μM oligomycin (ATP synthase inhibitor), with 3.5 μM carbonyl cyanide-p-trifluoromethoxy phenylhydrazone (FCCP), and finally, with 0.5 μM rotenone/antimycin A.

### Quantification of Gene Expression Assay

Quantification of gene expression was performed by ABI 7500 Sequence Detection System (Applied Biosystems, Foster City, CA, United States). The qPCR assays were performed as described in our previous study (Chen et al., 2016). The primers used for qPCR are shown in Supplementary Table 1. The mRNA levels were determined by qPCR in triplicate for each of the independently prepared RNA samples, and mRNA levels were normalized against the levels of glyceraldehyde 3-phosphate dehydrogenase (*GAPDH)* expression.

### Western Blotting Analysis

Total protein was extracted from astrocytes or brain tissue and quantified by bicinchoninic acid (BCA) protein assay kit (Pierce, United States) and used for immunoblotting analysis, as described previously. Briefly, the blot was incubated with a specific primary antibody overnight (anti- GFAP, 1:1,000), followed by incubation with HRP-conjugated secondary antibody. The bands were detected with a chemiluminescence detection kit (Millipore Co., MA, United States) and scanned using the iBright FL1500 chemiluminescence imaging system (Thermo Fisher Scientific, United States).

### Statistical Analysis

The results are presented as the mean ± standard error of the mean (SEM). For comparisons between two or multiple groups, the Student’s *t*-test or one-way analysis of variance (ANOVA) analysis was conducted, respectively. Significance is indicated by asterisks: *p < 0.05, ^**^p < 0.01, ^***^p < 0.001.

## Results

### Repeated Methamphetamine Administrations Induce Anxiety-Like Behavior in Mice

**FIGURE 1:**
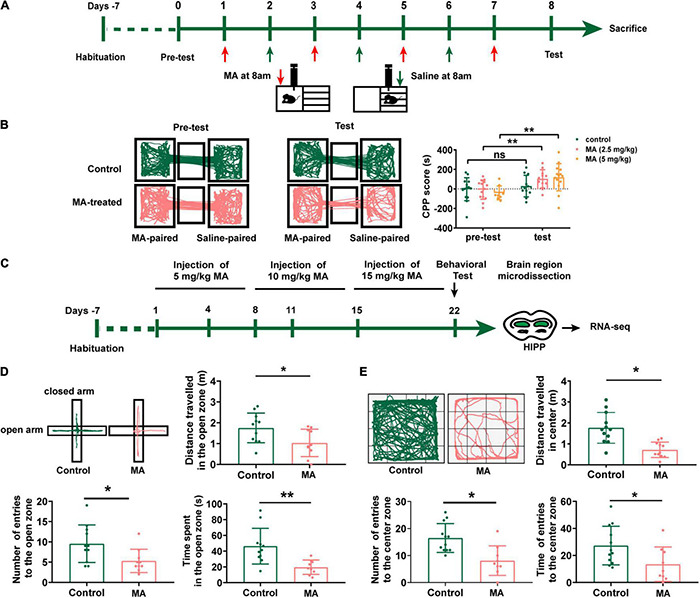
Repeated MA administrations induce anxiety-like behavior in mice. **(A)** Timeline of the Conditional place preference (CPP) experimental procedure. **(B)** The movement trajectory of the two groups of mice across compartments (left); CPP scores were assessed as the difference of time spent in the drug-paired compartment between the post and pre-conditioning phases (right). Data collected from three independent experiments. **(C)** Timeline of the model establishment and sample collection. **(D,E)** Anxiety assessment with the elevated plus maze (EPM) and open field test (OFT). Student *t*-tests, *p < 0.05 and **p < 0.05. Data collected from three independent experiments.

Initially, to identify an optimal concentration for the generation of an MA-addicted mouse model, we tested three different MA concentrations (2.5, 5, and 10 mg/kg, i.p.). Mice were trained to associate the MA reward with the paired context during training for the MA CPP (Figure 1A). A preference for the MA-paired side indicates the expression of a reward–context associated memory, which was assessed by measuring the time that an animal spent on the MA-paired side in the CPP apparatus. After four sessions of MA CPP training, mice treated with 5 mg/kg MA as well as the 2.5 mg/kg MA-treated group (Figure 1B, *p* < 0.05), respectively, showed a significant preference for the MA-paired side. However, 6 of 8 mice died in the 10 mg/kg MA-treated group. We then adopted 5 mg/kg MA as the initial concentration to establish a subsequent mouse model (Figure 1C), due to 2.5 mg/kg MA-group failing to exhibited anxiety-like behaviors (data not shown).

After 3 weeks with escalating dose of MA treatment, mice demonstrated a fear of entering the open arms of the EPM test (Figure 1D) with fewer numbers of entries to open zone and less time (*p* < 0.01) and shorter distance (*p* < 0.05) spent in the open zone. Consistently, MA treatments also displayed decreased locomotor activity and spent significantly less time in the center of the OFT than control mice (Figure 1E, *p* < 0.01). This suggested we successfully generated an animal model with anxiety-like behaviors.

### RNA-seq Revealed Differentially Expressed Genes in the Hippocampus in the Methamphetamine-Treated Mouse Model

**FIGURE 2:**
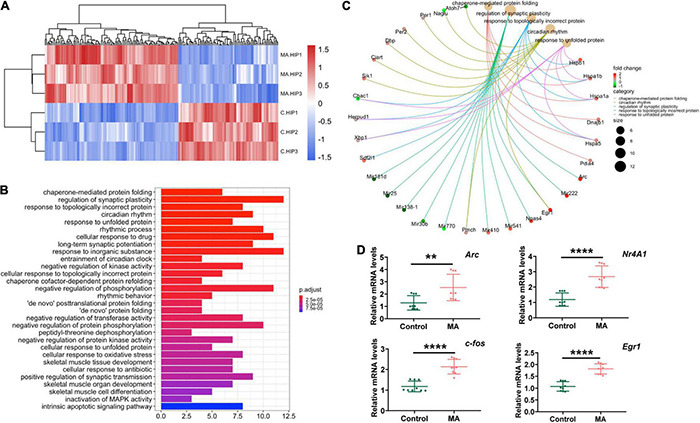
RNA-seq revealed differentially expressed genes in the HIPP in the MA-treated mouse model. **(A)** Hierarchical clustering analysis of RNAs with altered expression between the two groups (*p* < 0.05, fold change > 2). Red strip, high relative expression; blue strip, low relative expression. Color intensity reflects the degree of expression increase or decrease. **(B)** Thirty most enriched KEGG classifications of assembled differential genes in HIPP. **(C)** The genes implicated by the Top 5 KEGG signaling pathways in HIPP. **(D)** qPCR validation for RNA-seq data.

To better elucidate the molecular features of MA-induced anxiety-like behaviors in mice, we performed RNA-seq to investigate DEGs between control and chronic MA-treated mice in HIPP. Hierarchical clustering analysis was performed and displayed in Figure 2A. Fourteen upregulated genes were identified between the control and MA-treated groups, which were displayed in Supplementary Table 2.

To further investigate the altered signaling pathways involved in mice presenting with MA-induced anxiety-like behavior, we then performed KEGG enrichment analyses. The results demonstrated that DEGs between the control and MA-treated groups were mainly enriched in following signaling pathways: protein folding (chaperone-mediated protein folding, response to unfolded protein, chaperone cofactor-dependent protein refolding, “*de novo*” posttranslational protein folding, “*de novo*” protein folding, cellular response to unfolded protein), synaptic plasticity and synaptic transmission (regulation of synaptic plasticity, positive regulation of synaptic transmission), rhythmic processes (regulation of synaptic plasticity, positive regulation of synaptic transmission), protein phosphorylation (negative regulation of phosphorylation, negative regulation of protein phosphorylation, peptidyl-threonine dephosphorylation), oxidative stress, and the intrinsic apoptotic signaling pathway (Figure 2B). The targeted genes related to the top 5 shared KEGG pathways are shown in Figure 2C. These pathways indicates mitochondria, which participated in protein folding, and synaptic plasticity and synaptic transmission, oxidative stress, and the intrinsic apoptotic (Cheng et al., 2015; Fang et al., 2015; Song et al., 2017; Saito and Imaizumi, 2018), plays an important role in MA-induced anxiety and might be promising therapeutic target.

Furthermore, as immediate early genes (IEGs) have been reported to be involved in MA use and the development of neuropsychiatric disorders (McCoy et al., 2011; Gallitano, 2020), we used three samples from each group to validate four selected DEGs which were classified as IEGs (*Fos, Egr1, Arc* and *Nr4a1*). The results revealed that, in general, MA upregulated all the four IEGs identified, in accordance with the RNA-seq results (Figure 2D).

### Administration of Quercetin Ameliorates Anxiety-Like Behaviors in a Methamphetamine Mouse Model

**FIGURE 3:**
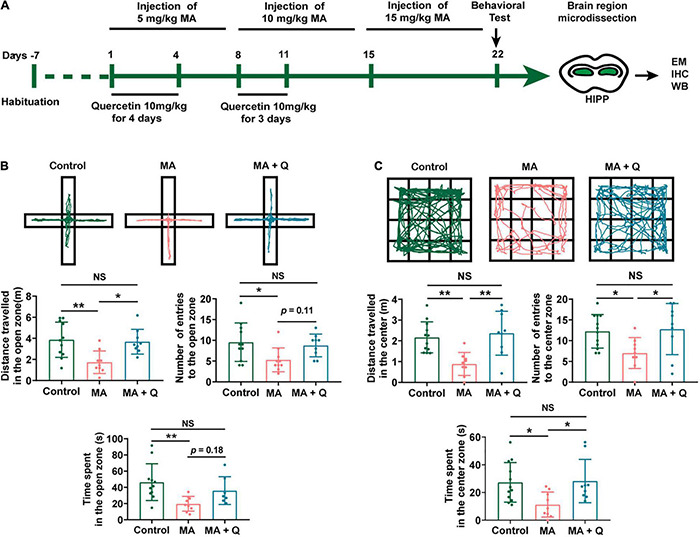
Administration of quercetin ameliorates anxiety-like behaviors in an MA mouse model. **(A)** Timeline of the model establishment and sample collection. **(B,C)** Anxiety assessment with the elevated plus maze (EPM) and open field test (OFT). Student *t*-tests, *p < 0.05 and **p < 0.05. Data collected from three independent experiments.

Quercetin has been reported to mitigate anxiety-like symptoms in a lipopolysaccharide-induced mouse model of anxiety (Samad et al., 2018; Lee et al., 2020) and has been reported as a mediator of mitochondrial function and ER stress (Khan et al., 2016). Therefore, we evaluated the effects of quercetin on MA-induced behavioral phenotypes and the role played by quercetin in mitochondrial functional modifications in the present study (Figure 3A).

In the EPM test, the time spent and the number of entries into the open arms were significantly reduced in the MA-treated group compared with those in the control group (Figure 3B, *p* < 0.05); in contrast, when compared with the control group, the time spent and number of entries into the closed arms were significantly elevated in the MA-treated group (Data not shown). However, mice treated with MA + Q showed the significant restoration of the time spent and the number of entries into the open arms compared with those for the single MA-treated group (Figure 3B, *p* < 0.05), with no significant differences observed between the control group and the MA + Q group (Figure 3B, *p* > 0.05).

The OFT results revealed that a single administration of MA significantly reduced the number of times MA-treated mice crossed in the central zone compared to the saline-treated group (Figure 3C, *p* < 0.05), while no significant difference in the number of crossings in the peripheral zone were observed (Data not shown). Quercetin combined with MA treatment significantly enhanced the number of central zone crossings compared with the single MA-treated group (Figure 3C, *p* < 0.05).

### Quercetin Ameliorates Mitochondrial Dysfunction and Aberrant Morphology in Astrocytes

Astrocytes have been shown to participate in anxiety development through the synaptic pruning of neurons (Çalışkan et al., 2020) and, although most of previous researches have focused on MA-induced neuronal injury. In this study, we performed experiments on astrocytes to explore its roles in MA-induced anxiety.

**FIGURE 4:**
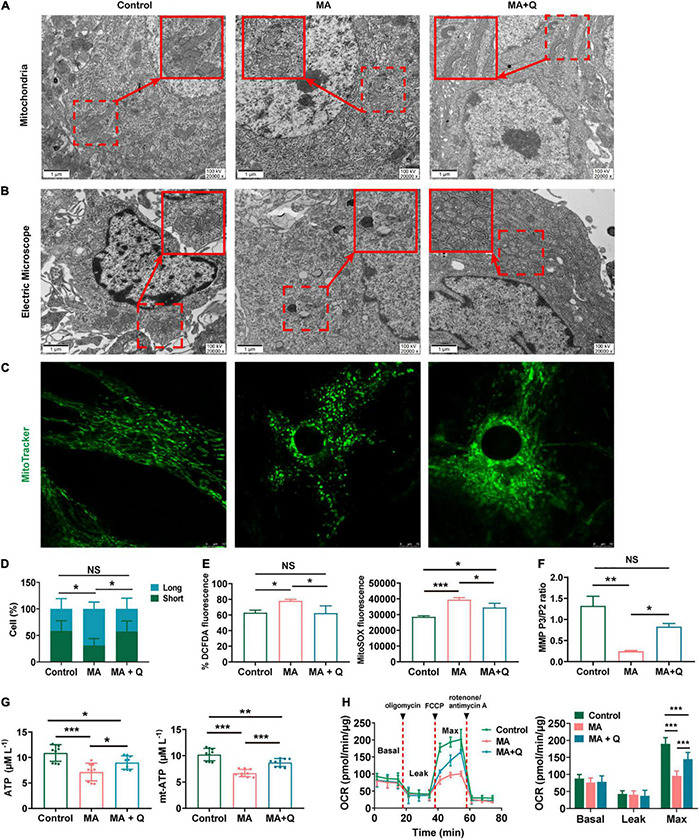
Quercetin ameliorates mitochondrial dysfunction and aberrant morphology in astrocytes. **(A)** Electron microscopy analysis (magnification, × 20,000) of astrocytes in HIPP. Areas in red boxes are magnified (Insets). **(B)** Electron microscopy analysis (magnification, × 20,000) in astrocytes. Areas in red boxes are magnified (Insets). **(C)** Representative images of astrocytic mitochondria. Alive astrocytes were incubated with MitoTraker Green as a probe for mitochondria in each group. **(D)** The percentage of astrocytes for short-shape (blue) and long-shape (green) mitochondria in different group is presented as histograms in panel **(C)**. **(E)** Total ROS (left) and mitochondria-derived ROS (right) production quantification by flow cytometry in each group. **(F)** Quantification of the mitochondrial membrane potential (MMP). **(G)** Quantification of the total (left) and mitochondria-derived ATP (right) by using an ATP quantification kits. **(H)** An analysis of O2 consumption in astrocytes. The Agilent SeahorseXFe24 analyzer measures OCR at basal and after injection of oligomycin (3.5 μM), FCCP (4 μM), and antimycin A (1 μM)/rotenone (1 μM) for three measurement cycles at each step (left). Basal, ATP-linked, maximal, and reserve capacity OCR in each group. *p < 0.05, **p < 0.01, and ***p < 0.001 vs. control, as determined by Student’s *t* test.

As signaling pathways linked to mitochondrial function have emerged as deregulated pathways forementioned, we also evaluated mitochondrial morphology of astrocytes in the HIPP using electron microscopy. The control group presented with relatively long, tubular mitochondria, whereas the mitochondria of MA-treated group were relatively more fragmented and swollen, with the loss of cristae (Figure 4A), indicating MA-induced damage to mitochondria of astrocytes in HIPP. Quercetin treatment was able to reinstate mitochondrial morphology toward long tubular mitochondria with intact cristae in MA-treated astrocytes (Figure 4A).

To further assess the effect of MA on mitochondria *in vitro*, we first evaluated mitochondrial morphology. The results demonstrated that tubular mitochondria in astrocytes shortened in length and swelled in width, forming large, spherical structures (Figure 4B), when exposed to MA. Similar morphological changes in mitochondria were viewed using mitotracker (Figure 4C). Quercetin treatment rescued abnormal mitochondrial morphology in astrocytes treated with MA (Figures 4A–C). The percentage of astrocytes with short/long mitochondria among different groups was analyzed and shown in Figure 4D.

Then, we test mitochondrial function in astrocytes. Mitochondria, as the intracellular source of ROS in animal cells, when confronting with oxidative stress, is the primary target attacked by ROS, and also produces excessive amounts of ROS due to the damage to enzymes in the electron transport chain. Therefore, we evaluated the total ROS by DCFDA and the mitochondrial ROS by MitoSOX staining respectively, and quantified the fluorescence intensity in MA-treated astrocytes with or without quercetin treatment. The results showed that both the total ROS (Figure 4E left, *p* < 0.05) and mitochondrial ROS (Figure 4E right, *p* < 0.001) were markedly increased in MA treated group, which were rescued by quercetin (Figure 4E, *p* < 0.05).

As we know, a series of redox reactions creates an electrochemical gradient through the mitochondrial electron transport chain, which drives the synthesis of ATP and generates the MMP. Therefore we measured the total and mitochondria-derived ATP, and MMP to evaluated mitochondrial function. The results revealed decreasing in MMP (Figure 4F, *p* < 0.01) and ATP (Figure 4G, *p* < 0.001) in MA-treated astrocytes, indicating mitochondria dysfunction after MA treatment. The quercetin supplementation of MA-treated astrocytes markedly elevated both the total ATP (Figure 4G left, *p* < 0.05) and mitochondria-derived ATP levels (Figure 4G right, *p* < 0.001) and increased MMP in MA-treated astrocytes (Figure 4F, *p* < 0.05). We used an XF24 metabolic bioanalyzer to assess the effects of quercetin supplementation on OCR in MA-treated astrocytes; as a result, we found that quercetin markedly reinstated the decreasing in the OCR induced by MA (Figure 4H, *p* < 0.001).

Considering mitochondrial also play an important role in neurons, we also evaluated the effects of quercetin on mitochondrial morphology and function in PC12. The mitochondria in MA-treated PC12 exhibited short and fragmented morphology with a significant decrease in number of PC12 (Supplementary Figures 1A,B) as compared to the control group. The quercetin supplementation partially restores mitochondrial morphology and the number of PC12 when exposed to MA. The results of mitochondrial function revealed that MMP (Supplementary Figure 1C, *p* < 0.05) and ATP (Supplementary Figure 1D, *p* < 0.05) decreased in 2 mM MA-treated PC12, accompanying an increase in ROS (Supplementary Figure 1E, *p* < 0.05). Quercetin diminished total ROS production (Supplementary Figure 1E, *p* < 0.05), but failed to rescue the abnormality of ATP production and MMP induced by MA (Supplementary Figures 1C,D, *p* > 0.05).

### Quercetin Mitigated Astrocytes Activation and Neuroinflammatory Induced by Methamphetamine

**FIGURE 5:**
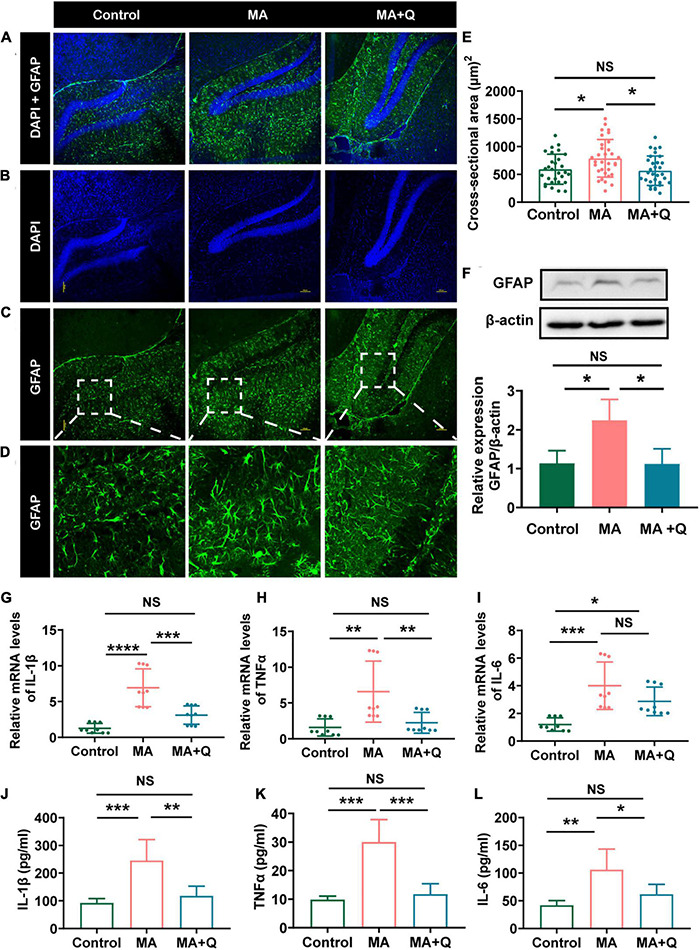
Quercetin mitigated astrocytes activation and neuroinflammatory induced by MA. **(A)** Merged image of panels **(B,C)**; Immunofluorescence was performed with anti-GFAP [green, **(C)**] and DAPI [blue, **(B)**]; **(D)** Enlarged images of the areas marked in panel **(C)** with a white box; **(E)** Area of astrocytes (μm^2^/cell) in each group, *p < 0.05, **p < 0.01. **(F)** Representative band pattern of the WB of different treatment of astrocytes using antibodies for GFAP and β-Actin (left); summary bar graphs of GFAP and β-Actin levels in different group (right). **(G–I)** Expression of proinflammatory factors by qPCR in the hippocampus (IL-1β, TNFα, IL-6). **(J–L)** The levels of the proinflammatory factors IL-1β were detected by ELISA. All experiments represent the average of 3 independent experiments.

Research have reported that astrocytes were activated in an MA-treated animal model, resulting in morphological and phenotypic abnormality (Zhou et al., 2019), and fragmented and dysfunctional mitochondria trigger A1 astrocytic response and propagate inflammatory neurodegeneration (Joshi et al., 2019). We evaluate astrocytes activation by immunostaining and western blotting for GFAP. The results indicated GFAP expression increased after MA treatment (Figure 5F, *p* < 0.05) with a larger area of astrocytes (Figures 5A–E, *p* < 0.05). Quercetin alleviated the activation of astrocytes (Figures 5A–F, *p* < 0.01), suggesting that MA treatment facilitated astrocytes activation, whereas quercetin reversed the cellular phenotypes.

Previous studies have shown that mitochondrial dysfunction in astrocytes, combining with neuroinflammation, impair the generation of reactive astrocytes and enhance neuronal cell death (Fiebig et al., 2019). Therefore, we assessed the change of neurons and microglia, which was considered as the main components innate neuroimmune cell, by immunostaining for NeuN and Iba-1. The results demonstrated MA administration markedly decreased the numbers of neurons (Supplementary Figures 2A–C) and promoted microglia proliferation (Supplementary Figures 2D–F).

Astrocytes and microglia, as key regulators of neuroinflammation in CNS, are activated during neuroinflammation to secrete inflammatory cytokines and chemokines (Kwon and Koh, 2020); consequently, we analyzed the expression of elevation of interleukin (IL)-1β, IL-6, and tumor necrosis factor (TNF)α, the pro-inflammatory cytokines which were triggered by drug abuse (Dang et al., 2021). As suspected, MA treatment accelerated astrocyte activation, accompanied by the elevation of IL-1β (Figure 5G, *p* < 0.001), TNFα (Figure 5H, *p* < 0.01), and IL-6 (Figure 5I, *p* < 0.001) in the HIPP and MA-treated astrocytes (Figures 5J–L). Quercetin, as an anti-inflammatory agent, decreases all the three cytokines *in vitro*, and attenuated the expression of IL-1β and TNFα, but not IL-6 in HIPP.

## Discussion

In the present study, we found mitochondrial morphological defects accompanied the dysfuntion in MA-treated mice with anxiety-like behavior. Quercetin attenuated anxious symptoms and pathology, and modified mitochondria and neuroinflammation in both a mouse model and cultured astrocytes treated with MA. These findings, for the first time, suggested that quercetin could inhibit the progression of MA-induced anxiety by modulating mitochondrial function and morphology and mitigating neuroinflammation in the central nervous system. The results indicated that quercetin represents a potential therapeutic medicine for future development and supported the hypothesis that changes in mitochondrial function mediate anxiety progression.

We found that MA-induced anxiety-like behavior in mice, as previously described (Ru et al., 2019; Jayanthi et al., 2021). Consistent with our findings, Iwazaki et al. (2008) have demonstrated synaptic plasticity- and synaptic transmission-, oxidative stress-, and intrinsic apoptotic-related signaling pathway were dysregulated in protein expression level in MA-treated rats. As an organelle generating secondary ROS, which can disturb protein folding and cause mitochondrial DNA mutations, mitochondria are regarded as both a source and a target of oxidative stress (Song et al., 2017), besides, they play an important role in apoptosis *via* the intrinsic apoptotic program which was identified to be dysregulated in frontal cortex in MA-treated rats (Iwazaki et al., 2008). Furthermore, ER-misfolded proteins have been reported to accumulate at ER-mitochondria contact regions, where they eventually imported into mitochondrial matrix and resulted in impaired mitochondrial function (Saito and Imaizumi, 2018; Cortés et al., 2021), and dysfunction mitochondria failed to maintain synaptic ion homeostasis and synaptic plasticity due to decreased ATP production and overloaded Ca^2+^ concentrations (Fang et al., 2015). In summary, MA exposure facilitated mitochondrial damage, intracellular ROS production (Cao et al., 2017), the depolarization of MMP and metabolic disturbance (Li et al., 2015), eventually leading to mitochondrial dysfunction (Xie et al., 2016) and intrinsic apoptotic program activation (Iwazaki et al., 2008). In this article, pathways related to mitochondria were markedly changed, and mitochondria were found to be short, fragmented with abnormal morphology after MA treatment *in vivo* and *in vitro*. Multiple parameters about mitochondria function were then displayed aberrant, such as ROS and ATP production, MMP, and OCR, implying that mitochondria as a target organelle in MA-treatment. Based on these, we speculate that mitochondria represent a primary target organelle of MA treatment in the HIPP.

Accumulating data has highlighted the contributions of brain mitochondria and bioenergetics to the development of psychiatric disorders and stress-related pathologies (Einat et al., 2005; Manji et al., 2012). The damaged mitochondria accumulation induced synaptic loss, and neuron apoptosis (de la Mata et al., 2017), underscoring the importance of mitochondria in the development of psychotic disorders. In addition, The fundamental role played by mitochondria in the synthesis of the primary excitatory neurotransmitter (glutamate) and the inhibitory neurotransmitter (γ-aminobutyric acid, GABA) suggest that the mitochondrial adaptations observed in the context of anxiety may contribute to an imbalance in neural excitation and inhibition, which is thought to underlie several neuropsychiatric disorders (Filiou and Sandi, 2019). Mitochondria-targeting drugs have been reported to show good outcomes in anxiety-related studies in both humans and rodents (Filiou and Sandi, 2019). In this article, we further demonstrated that modification of mitochondrial function and morphology might represent a potential strategy for alleviating anxiety-like behaviors induced by MA, since chronic MA exposure resulted in the dysregulation of mitochondrial morphology and mitochondrial dysfunction. Quercetin, as a bioactive compound with diverse pharmacologic effects, has been reported to exert several beneficial effects, including neuroprotective effects (Costa et al., 2016), the regulation of the sleep–wake cycle (Kambe et al., 2010), and the optimization of mitochondrial function (Houghton et al., 2018). In this study, quercetin supplementation significantly mitigated MA-induced anxiety-like behavior by improving mitochondrial morphology and function, alleviating neuronal injury, both *in vivo* and *in vitro*. Here, we also identified the novel pharmacological efficacy of quercetin in the treatment of anxiety induced by MA.

As a major source of glycogen and lactate, astrocytes provided neurons with additional energy and play key metabolic roles in the CNS (Shirakawa et al., 2010). Moreover, astrocytes can dispose of and recycle damaged mitochondria released by neurons, and release healthy extracellular mitochondrial particles to neuron (Hayakawa et al., 2016). Astrocyte dysfunction has been shown to facilitate the pathogenesis of neurological and psychiatric disorders (Tian et al., 2018). Hence, astrocytes and the mitochondria may represent an important target of neurological and psychiatric disorders. Thus, we intended to focus this study on astrocytes in subsequent experiments. Research has shown MA exposure facilitated dysfunction and morphological abnormalities of mitochondrial (Valian et al., 2019), which can trigger various innate immune signaling pathways in a cell-intrinsic or -extrinsic manner (Bader and Winklhofer, 2020), resulting in the release of inflammatory factors. In this work, we observed MA-induced morphological abnormalities of mitochondria *in vivo* and *in vitro*, and mitochondrial dysfunction in astrocytes, and the phenotypes were rescued by quercetin. As we know, except for microglia, astrocytes also serve as crucial regulators of the innate and adaptive immune responses, and play critical roles in neuroinflammation (Colombo and Farina, 2016). In this study, we found that MA treatment accelerated astrocyte activation, accompanied by the elevation of interleukin (IL)-1β, IL-6, and tumor necrosis factor (TNF)α in the HIPP and MA-treated astrocytes (Figure 5). Quercetin, as a bioactive compound with antioxidant and anti-inflammatory properties (Khan et al., 2016), reduced the expression of IL-1β and TNFα *in vitro* and *in vivo*, and alleviated astrocytes activation when treated with MA in this work (Figure 5). These results suggested that MA treatment accelerated astrocytes activation and facilitated the release of inflammatory factors, which can trigger neuronal apoptosis and synaptic loss (Garwood et al., 2011; Çalışkan et al., 2020). Moreover, the excessive accumulation of damaged mitochondria, combined with astrocyte activation and the increase in inflammatory factors, induces neurotoxicity and promotes neuronal apoptosis (Nicholls, 2004), which initiates a vicious cycle that aggravates the anxiety process (Garwood et al., 2011; Çalışkan et al., 2020). These findings also confirmed the neuroprotective activity of quercetin, through modulating astrocytes and rebalancing neuroinflammation levels activation.

Nevertheless, the mechanisms by which quercetin exerts its anti-neuroinflammation activity and modulates mitochondrial function remain unclear. According to previous reports, Nr4a1 (also known as TR3 or NGFI-B), which was upregulated in our RNA-seq results, is an orphan member of the nuclear receptor superfamily. It migrates from the nucleus to the mitochondria, where it binds to Bcl-2 to induce apoptosis and cause the release of cytochrome c (Liu et al., 2008). Nr4a1 evoked cellular oxidative stress and disrupt ATP generation (Zhang and Yu, 2018), and the expression and activity of Nr4a1 are sustained by chronic stress in animal models and in human studies of neuropathologies sensitive to the buildup of chronic stress (Akiyama et al., 2008; Jeanneteau et al., 2018). Nr4a1 has also been shown to be important for regulating metabolic and morphological aspects of neuronal functions by modifying the expression of several mitochondrial regulatory genes, including *Mfn1*, *Mfn2, Fis1* and *OPA1* (Jeanneteau et al., 2018), which can result in mitochondrial dysfunction and behavioral phenotypes. Therefore, we speculated that quercetin might modulate genes associated with mitochondrial function *via* downregulating Nr4a1. Thus, we detected the expression of Nr4a1 in MA + quercetin and MA groups *in vivo*, the results verified the effects of quercetin in decreasing the Nr4a1 expression both in RNA and protein level (Supplementary Figure 3). Future work will explore the molecular mechanism extensively.

In conclusion, our study indicated that MA cause damage to brain cells, including neurons and astrocytes, by influencing mitochondrial metabolism, energy production, and morphology in astrocytes, which participated in the development of neuropsychiatric disorders, such as anxiety. The oral supplementation of quercetin neutralized the neuropsychiatric status of MA-treated mice; our findings indicated the potential of quercetin as a candidate agent for alleviating MA-induced anxiety and support the hypothesis that mitochondria mediate anxiety progression. Further study is necessary to illustrate the contributions and mechanisms of mitochondria in the progression of anxiety induced by MA.

## Data Availability Statement

The raw reads data from mRNA-seq are available at NCBI GEO with the accession number: GSE193829 and Supplementary Material, further inquiries can be directed to the corresponding authors.

## Ethics Statement

The animal study was reviewed and approved by Committee on Ethics in the Use of Animals from Kunming Medical University.

## Author Contributions

FC and JY designed the experiments and participated in the data analysis. FC, JY, QW, and YL wrote the manuscript. JS and CC performed the sequence alignment and bioinformatics. MC, YZ, and WT performed the animal experiments. FC, LZ, and ZZ performed the cell experiments. HW, HL, YX, and MZ participated in data analysis. JY and KW conceived of the study and supervised the project. All authors read and approved the final manuscript.

## Conflict of Interest

The authors declare that the research was conducted in the absence of any commercial or financial relationships that could be construed as a potential conflict of interest.

## Publisher’s Note

All claims expressed in this article are solely those of the authors and do not necessarily represent those of their affiliated organizations, or those of the publisher, the editors and the reviewers. Any product that may be evaluated in this article, or claim that may be made by its manufacturer, is not guaranteed or endorsed by the publisher.

## Footnotes

1
http://metascape.org

## Supplementary Material

Supplementary Figure 1 The effect of quercetin on mitochondrial morphology and function in MA-treated in PC12. **(A)** Representative images of mitochondria in PC12. Alive cells were incubated with MitoTraker Green as a probe for mitochondria in each group. **(B)** Enlarged images of the areas marked in A with a white box. **(C)** Quantification of the mitochondrial membrane potential (MMP). **(D)** Quantification of the total ATP in PC12. **(E)** Total ROS production quantification by flow cytometry in each group. All experiments represent the average of 3 independent experiments. **p* < 0.05, as determined by Student’s *t* test.

Supplementary Figure 2 Confocal microscopic analysis of neuron and astrocytes using immunofluorescence stainings. **(A–C)** Immunofluorescence was performed with anti-NeuN [green, **(C)**] and DAPI [blue, **(B)**], **(C)** Merged image of panels **(A,B)**. **(D–F)** Immunofluorescence was performed with anti-Iba [green, **(D)**] and DAPI [blue, **(E)**], **(F)** Merged image of panels **(D,E)**. All experiments represent the average of 3 independent experiments.

Supplementary Figure 3 Quercetin rescued MA-induced Nr4a1 upregulation in gene and protein level. **(A)** Representative band pattern of the WB of different treatment of HIPP using antibodies for Nr4a1 and GAPDH. **(B)** Summary bar graphs of Nr4a1 and GAPDH levels in different groups in hippocampus. **(C)** Expression of Nr4a1 and GAPDH by qPCR in HIPP. **p* < 0.05, ^***^*p* < 0.0005, and ^****^*p* < 0.0001. All experiments represent the average of 3 independent experiments.
