# Supplementary figures and images for: Quercetin Mitigates Methamphetamine-Induced Anxiety-Like Behavior Through Ameliorating Mitochondrial Dysfunction and Neuroinflammation
Source: Front Mol Neurosci. 2022 Feb 28;15:829886. doi: 10.3389/fnmol.2022.829886 (PMC8919775; doi:10.3389/fnmol.2022.829886)

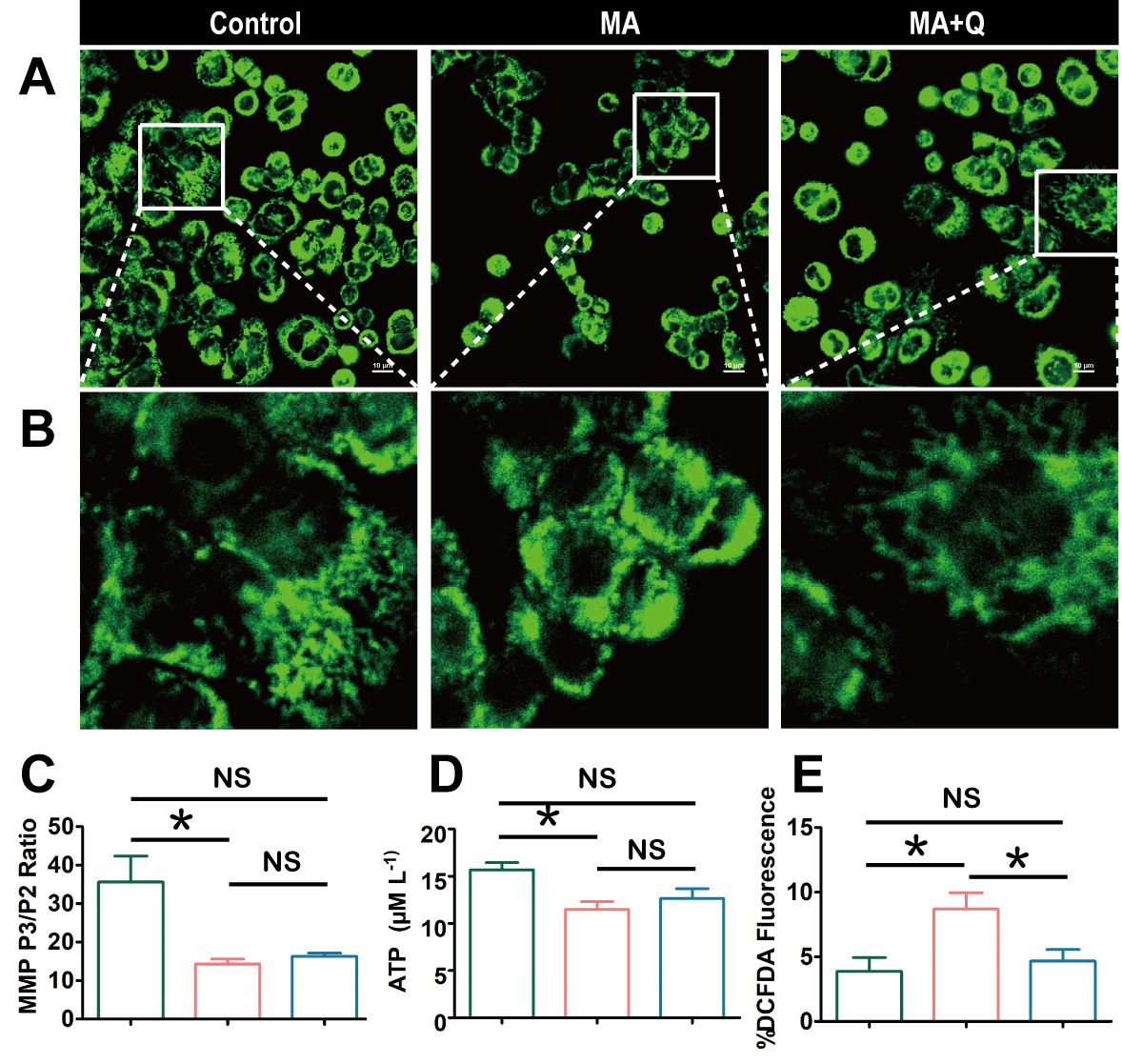

Supplement: Supplementary Figure 1 — The effect of quercetin on mitochondrial morphology and function in MA-treated in PC12. (A) Representative images of mitochondria in PC12. Alive cells were incubated with MitoTraker Green as a probe for mitochondria in each group. (B) Enlarged images of the areas marked in A with a white box. (C) Quantification of the mitochondrial membrane potential (MMP). (D) Quantification of the total ATP in PC12. (E) Total ROS production quantification by flow cytometry in each group. All experiments represent the average of 3 independent experiments. *p < 0.05, as determined by Student’s t test. [file Image_1.TIF]

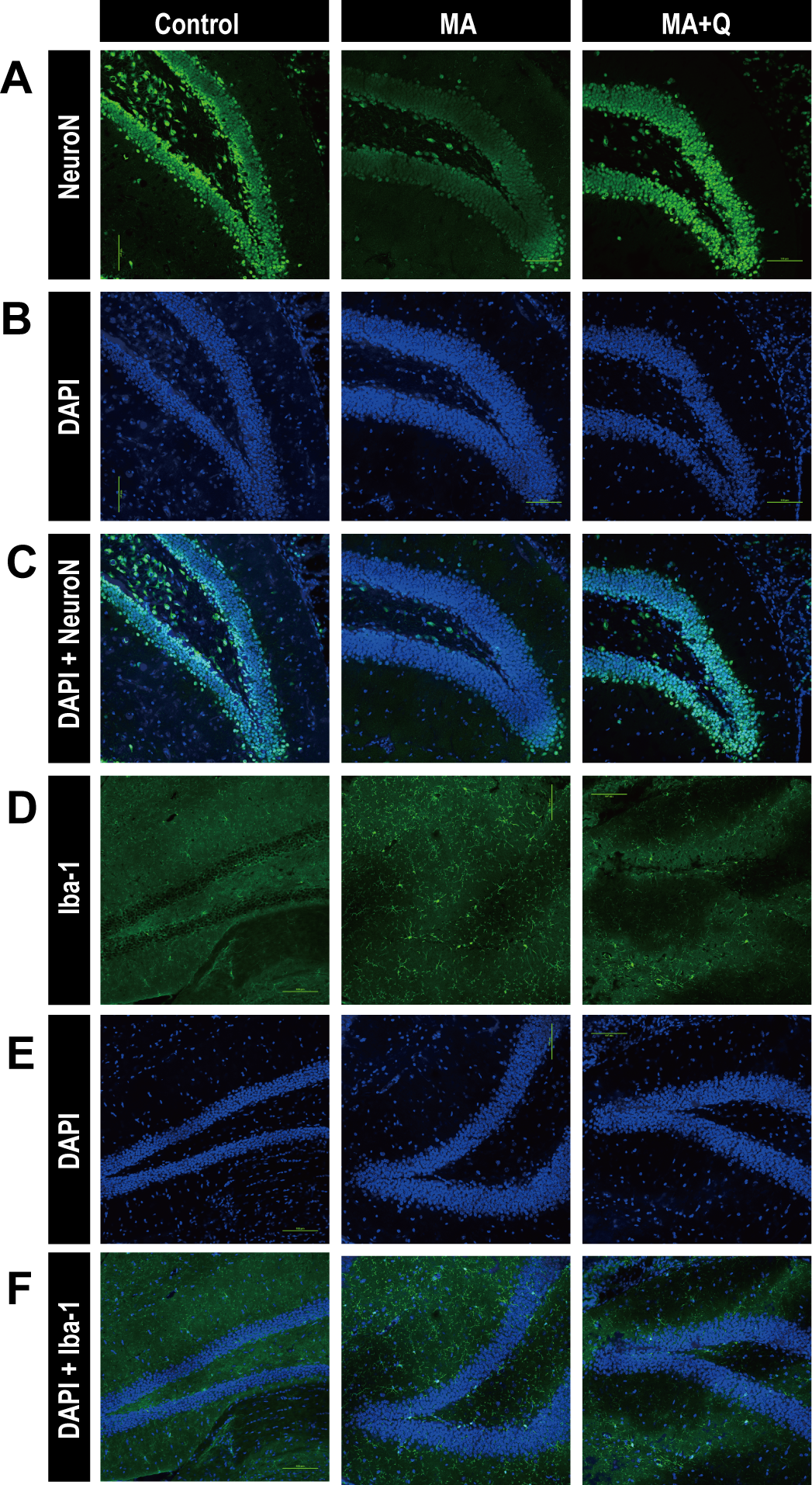

Supplement: Supplementary Figure 2 — Confocal microscopic analysis of neuron and astrocytes using immunofluorescence stainings. (A–C) Immunofluorescence was performed with anti-NeuN [green, (C)] and DAPI [blue, (B)], (C) Merged image of panels (A,B). (D–F) Immunofluorescence was performed with anti-Iba [green, (D)] and DAPI [blue, (E)], (F) Merged image of panels (D,E). All experiments represent the average of 3 independent experiments. [file Image_2.TIF]

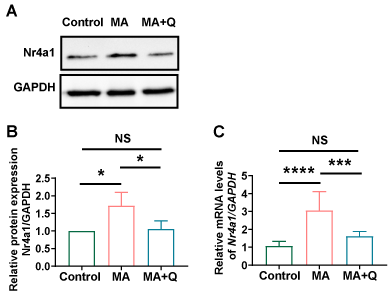

Supplement: Supplementary Figure 3 — Quercetin rescued MA-induced Nr4a1 upregulation in gene and protein level. (A) Representative band pattern of the WB of different treatment of HIPP using antibodies for Nr4a1 and GAPDH. (B) Summary bar graphs of Nr4a1 and GAPDH levels in different groups in hippocampus. (C) Expression of Nr4a1 and GAPDH by qPCR in HIPP. *p < 0.05, ***p < 0.0005, and ****p < 0.0001. All experiments represent the average of 3 independent experiments. [file Image_3.TIF]
